# Supplementary material for: Topological defects in multi-layered swarming bacteria
Source: Soft Matter. 2024 May 15;20(21):4237–45. doi: 10.1039/d4sm00038b (PMC11135144; doi:10.1039/d4sm00038b)
Supplement: SM-020-D4SM00038B-s002 [file SM-020-D4SM00038B-s002.pdf]

Electronic Supplementary Material (ESI) for Soft Matter.
This journal is © The Royal Society of Chemistry 2024

Supplementary Information: Topological defects in multi-layered swarming bacteria

Victor Yashunsky,[a] Daniel J.G. Pearce,[b] Gil Ariel,[c] Avraham Be'er[d,e]

[a] The Swiss Institute for Dryland Environmental and Energy Research, The Jacob Blaustein Institutes for Desert Research, Ben-Gurion University of the Negev, Sede Boqer Campus, 84990 Midreshet Ben-Gurion, Israel; E-mail: yashunsk@bgu.ac.il;

[b] Department of Theoretical Physics, University of Geneva, 1211 Geneva, Switzerland;

[c] Department of Mathematics, Bar-Ilan University, 52900 Ramat-Gan, Israel; E-mail: arielg@math.biu.ac.il;

[d] Zuckerberg Institute for Water Research, The Jacob Blaustein Institutes for Desert Research, Ben-Gurion University of the Negev, Sede Boqer Campus, 84990 Midreshet Ben-Gurion, Israel;

[e] The Department of Physics, Ben-Gurion University of the Negev, 84105 Beer-Sheva, Israel; E-mail: beera@bgu.ac.il.

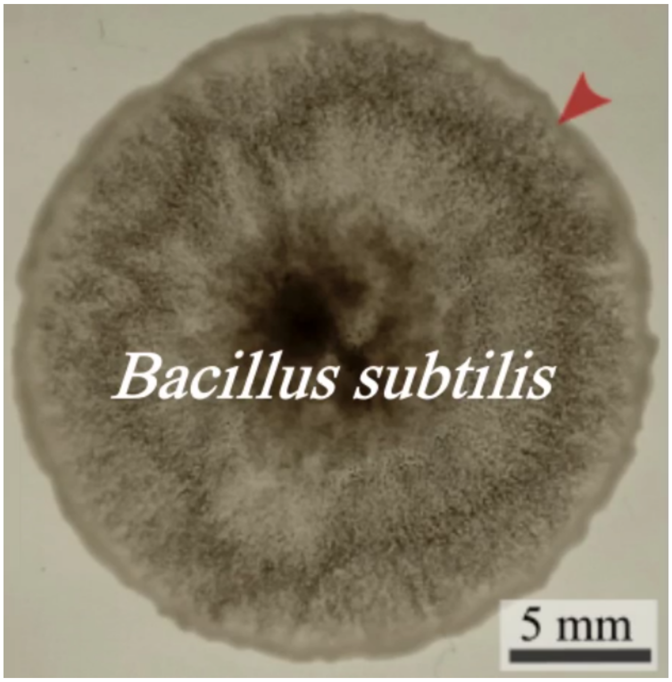

MOV. S1: *B. subtilis* swarming analysis. A top-view macroscopic image capturing the overall dynamics of a *B. subtilis* colony; High-resolution fluorescent microscopy focused on the region of interest, indicated by the red arrow; Extraction and visualization of the nematic director field from the obtained images; Tracking of half-integer defects, with $+1/2$ defects marked in red and $-1/2$ defects in blue, providing insights into the swarming behavior.

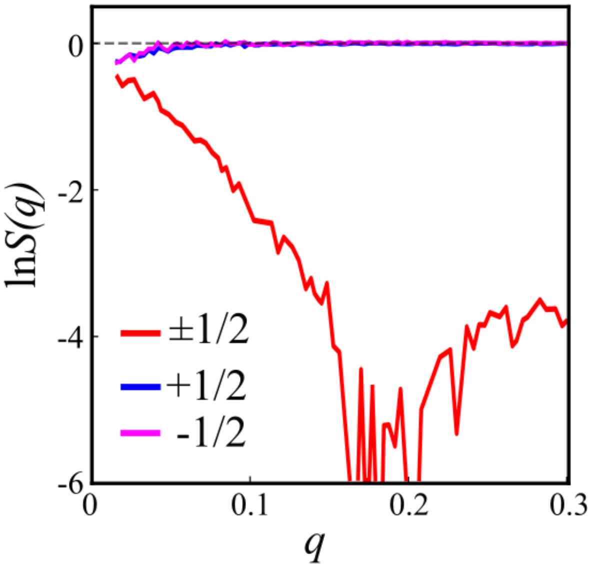

Fig. S 1 Defect structure factors. The structure factor for $+1/2$ defects on their own, $S_{+1/2}(q)$ as a function of the norm of the wave vector, q (blue) as well as the structure factor for $-1/2$ defects on their own, $S_{-1/2}(q)$ (magenta), are decreasing to zero as $q \to 0$, consistent with hyperuniformity. The structure factor derived from cross-correlations, $S_{-1/2}(q)$ (red) is small. See the methods section for definitions.

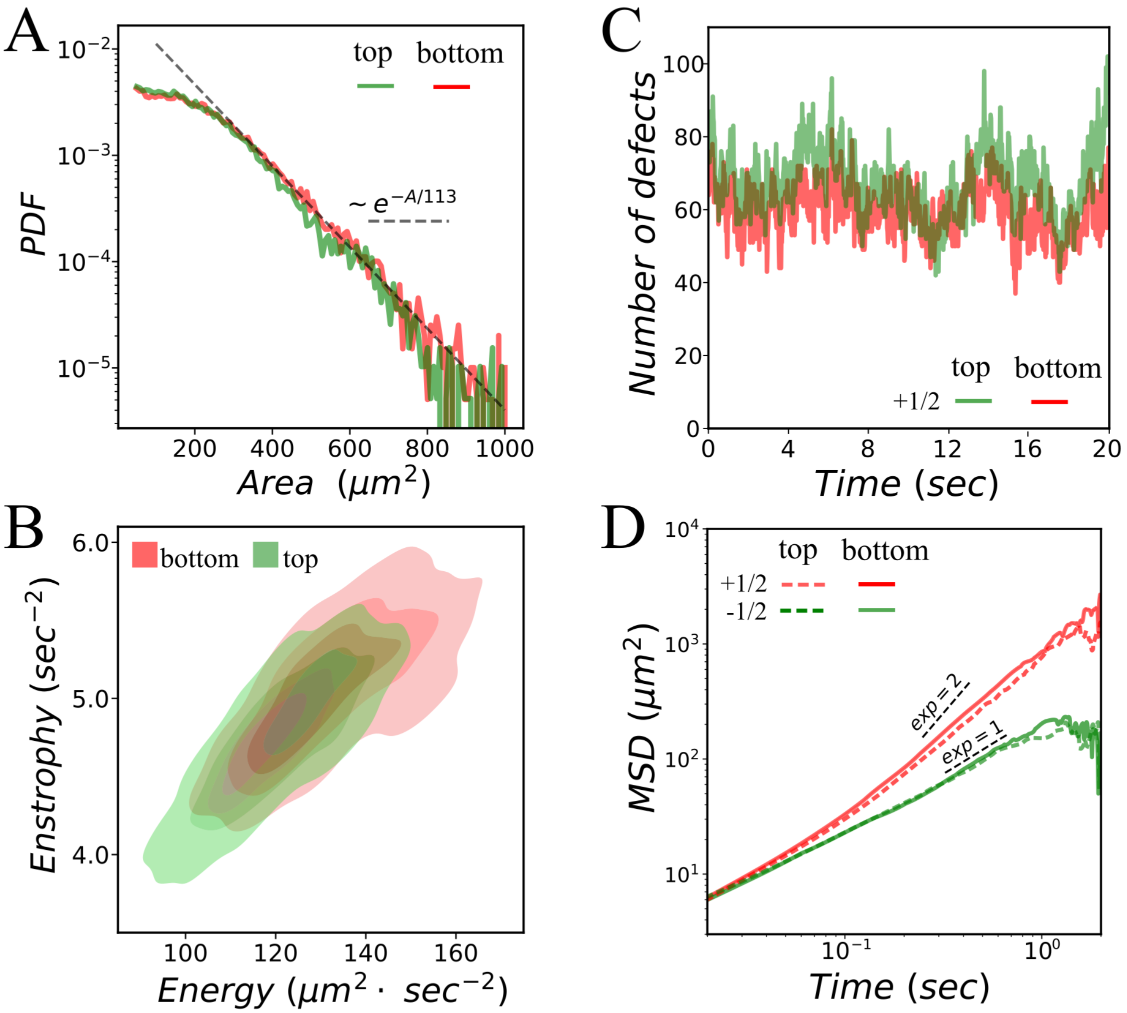

Fig. S 2 Comparison between the bottom and top layers of *B. subtilis* swarming: Statistical analysis of the director and velocity fields. (A) Log-linear representation of the vortex area probability density distribution in the bottom (red) and the top layer (green) of the colony. The dotted line shows an exponential fit. (B) Density plot of enstrophy vs. kinetic energy levels for each timepoint over the entire experiment. Red and green areas correspond to the bottom and the top layer of the colony. (C) Time evolution of the observed number of +1/2 defects in the bottom (red) and the top (green) layer of the colony. (D) Averaged mean square displacement (MSD) of +1/2 (red) and -1/2 (green) defects in the bottom (solid line) and the top layer (dashed line) of the colony. The black dashed lines are guides to the eye with slopes 1 (diffusive) and 2 (ballistic).
